# Composition of fungal soil communities varies with plant abundance and geographic origin

**DOI:** 10.1093/aobpla/plv110

**Published:** 2015-09-14

**Authors:** Vanessa Reininger, Laura B. Martinez-Garcia, Laura Sanderson, Pedro M. Antunes

**Affiliations:** 1 Department of Biology, Algoma University, Sault Ste. Marie, ON, P6A 2G4 Canada; 2 Present address: Agroscope, Institute for Plant Production Sciences IPS, Schloss 1, 8820 Wädenswil, Switzerland; 3 Present address: ETH Zurich, Institute of Integrative Biology, Forest Pathology and Dendrology, Universitätstrasse 16, 8092 Zurich, Switzerland; 4 Present address: Department of Soil Quality, Wageningen University, 6700 AA, Wageningen, The Netherlands

**Keywords:** Arbuscular mycorrhizal fungi, community ecology, α diversity, β diversity, 454 pyrosequencing

## Abstract

Interactions of belowground fungal communities with exotic and native plant species may be important drivers of plant community structure in invaded grasslands. However, field surveys linking plant community structure with belowground fungal communities are missing. We investigated whether a selected number of abundant and relatively rare plants, either native or exotic, from an old-field site associate with different fungal communities. We also assessed whether these plants showed different symbiotic relationships with soil biota through their roots. We characterized the plant community and collected roots to investigate fungal communities using 454 pyrosequencing and assessed arbuscular mycorrhizal colonization and enemy-induced lesions. Differences in fungal communities were considered based on the assessment of α- and β diversity depending on plant ‘abundance’ and ‘origin’. Plant abundance and origin determined the fungal community. Fungal richness was higher for native abundant as opposed to relatively rare native plant species. However, this was not observed for exotics of contrasting abundance. Regardless of their origin, β diversity was higher for rare than for abundant species. Abundant exotics in the community, which happen to be grasses, were the least mycorrhizal whereas rare natives were most susceptible to enemy attack. Our results suggest that compared with exotics, the relative abundance of remnant native plant species in our old-field site is still linked to the structure of belowground fungal communities. In contrast, exotic species may act as a disturbing agent contributing towards the homogenization of soil fungal communities, potentially changing feedback interactions.

## Abstract (precis)

We investigated whether abundant and relatively rare plants, either native or exotic, from an old-field site associate with different fungal communities and their symbiotic relationships with soil biota. Plant abundance and origin determined the fungal community. Fungal richness was higher for native abundant as opposed to relatively rare native plant species. This was not observed for exotics of contrasting abundance. Abundant exotics were the least mycorrhizal whereas rare natives were most susceptible to enemy attack. Our results suggest that compared to exotics, the relative abundance of native plant species in our old field-site is linked to the structure of belowground fungal communities.

## Introduction

One of the major goals in ecology is to understand why some plant species in natural communities are more abundant than others (Hubbell 2001; Chave 2004; McGill *et al*. 2007; Dumbrell *et al*. 2010). Many studies focusing on plant community ecology have provided insight into community assembly through testing plant–plant competitive interactions and plant–abiotic interactions such as nutrient and light availability (Tilman 1985; Thompson 1987; Wilby and Brown 2001; Miller *et al*. 2005; Dybzinski and Tilman 2007; Bever *et al*. 2012). There is increasing evidence that belowground plant–microbe interactions are important to explain plant community dynamics (Bever *et al*. 1997, 2010, 2012; Reynolds and Haubensak 2009). Many studies have already been conducted focusing on belowground microbes and their interactions with the host plant (Johnson *et al*. 1997; Jumpponen and Trappe 1998; Reynolds *et al*. 2003; Bonfante and Anca 2009; Compant *et al*. 2010; Porras-Alfaro and Bayman 2011; Reininger *et al*. 2012; Reininger and Sieber 2012, 2013). However, little is known about the impact of plant community structure on the belowground soil microbial community (Klironomos 2003; Reynolds *et al*. 2003; Bagchi *et al*. 2010; Bever *et al*. 2010, 2012) especially with regard to plant communities which are progressively invaded by exotic plant species (Levine 2000; Klironomos 2002). According to the Wild Species Report issued by the Government of Canada, extant natural plant communities in Canada now include a large portion of exotic species, comprising 24 % of all the vascular plants (Wild Species 2010, http://publications.gc.ca/collections/collection_2011/ec/CW70-7-2010-eng.pdf, November 2014). Due to establishment of exotic plant species, plant community structure can change and consequently, interactions with soil microbial communities may change accordingly. By comparing the associations between co-occurring native and exotic plant species and their respective belowground fungal communities, we may be better able to understand and predict plant community dynamics in invaded ecosystems.

According to Klironomos (2002), plant species relative abundance may partially be explained by interactions between plants and soil microbes. Plant–soil feedbacks are defined as the effect that plant species exert on soil biotic and abiotic factors, which in turn can feedback into growth and fitness effects on subsequent conspecific or heterospecific plants (Van der Putten *et al*. 2013). In native plants, feedbacks are subjected to co-evolution between symbionts. Exotic species invading a new range will also establish interactions with the soil biota but by virtue of the host plant's novelty in the system, those interactions may not yet have experienced co-evolutionary processes. As a result, exotics may associate indiscriminately with soil microbial communities and function as a non-host for some plant pathogens, i.e. they show a symptomless infection (Nürnberger and Lipka 2005; Schulz and Boyle 2005). These fungal symbionts, accumulating in exotic species without necessarily causing population declines, may in turn negatively affect native plants to which they are pathogenic (Roy and Handley 2012; Flory and Clay 2013; Van der Putten *et al*. 2013).

Exotic plants are assumed to leave their enemies behind in their home geographic range (Enemy Release Hypothesis) and this could explain the differential in feedback in their new range relative to natives, which are more affected by a negative feedback (Keane and Crawley 2002). However, ∼50 % of the studies do not support the Enemy Release Hypothesis (Agrawal *et al*. 2005; Jeschke *et al*. 2012). These contrasting hypotheses highlight the uncertainty surrounding microbial communities associating with native and exotic plant species in natural communities and how alterations imposed by exotics may affect the soil biota community and by extension, the entire plant community.

Next-Generation Sequencing technologies such as 454 pyrosequencing using universal primers provide an opportunity to survey the soil microbial diversity associated with plant species in natural communities (Buée *et al*. 2009; Costa and Antunes 2012). These surveys contribute to improve our understanding of the biodiversity of microorganisms that co-exist and interact with plant roots across spatial scales and possibly influence plants’ relative abundance and the dynamics between native and exotic plant species. Specifically, fungi are key drivers of primary productivity and plant community structure due to their diverse roles as mycorrhizal fungi, endophytes, decomposers and pathogens. They directly interact with plant roots resulting in functional outcomes that range from mutualism to parasitism (Schulz and Boyle 2005; Sinclair and Howard 2005; Smith and Read 2008). Better knowledge of fungal community structure within plant roots among different plant species is a first step towards understanding how fungi influence plant community dynamics.

We surveyed the fungal diversity present in the roots of four native and four exotic plant species from a natural old-field plant community. Within each of these two groups, two species were highly abundant in the community and the other two were relatively rare. α Diversity was used to measure the fungal diversity in each plant species whereas β diversity was a measure of variation in fungal composition among groups (abundant versus rare and native versus exotic) (Anderson *et al*. 2011). We also determined levels of arbuscular mycorrhizal colonization and lesions inflicted by enemies to plant roots.

We predicted that fungal communities associating with plant roots differ depending on plant species ‘abundance’ and ‘origin’. More specifically, abundant natives would associate with more similar fungal communities than rare natives as they are more often surrounded by conspecifics that are likely to harbor the same fungal community. However, exotics were expected to associate with a more variable fungal community as they are not yet restricted to a particular (i.e. co-evolved) fungal community. Abundant and rare exotic plant species were expected to show the same variable pattern as they have not had the same amount of time to interact with the belowground fungal communities and establish species-specific relationships in comparison to the natives.

## Methods

### Study site and plant community diversity

**Figure 1.**
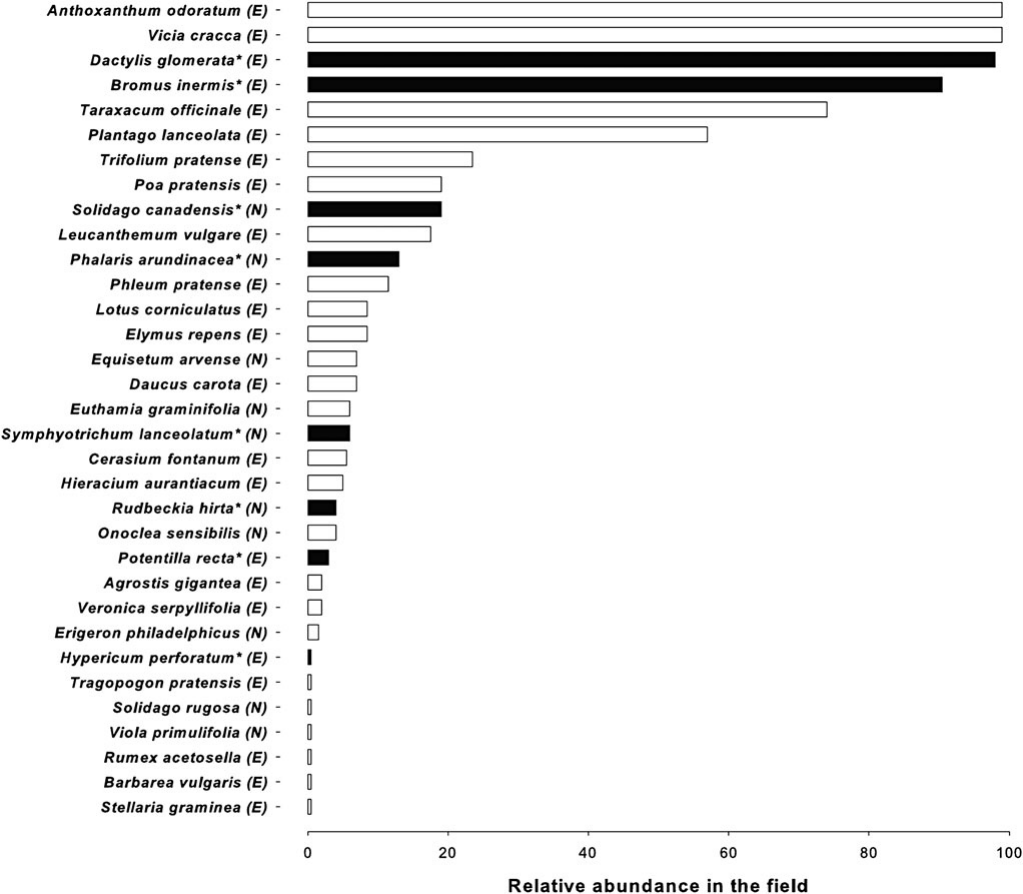
Plant community at the old-field site in Sault Ste. Marie, ON, Canada. Relative abundance was calculated as the number of plots in which each species was present out of 100 plots (1 × 1 m). Black bars and species names with an asterisk indicate the species used in this study. E, exotic origin; N, native origin.

The study site, our long-term ecological research site (LTERS), is a 100 × 100 m flat homogeneous (i.e. plant cover and edaphic conditions) old agricultural field at the Ontario Ministry of Natural Resources Research (OMNR) Arboretum in Sault Ste. Marie, Ontario (46°32′34.1″N, 84°27′29.4″W). The field site has not been cultivated for six decades and has not been mowed since 2009. The soil is a silty loam and soil analysis conducted in 2011 were as follows (*n* = 6): 22.6 ± 1.06 % clay, 53.1 ± 0.27 % silt, 24.4 ± 1.07 % sand, 0.13 ± 0.001 % total N, 1.81 ± 0.02 % total C, 11.4 ± 0.07 mg kg^−1^ Bray available P, 360.7 ± 2.55 mg kg^−1^ Ca, 45.0 ± 0.23 mg kg^−1^ K, 38.9 ± 0.32 Mg and pH = 5.74 ± 0.01. The field was divided into two hundred and eighty-nine 1 × 1 m plots on a 17 × 17 plot grid. Each plot was surrounded by a 1 m wide buffer zone. One hundred plots were selected randomly for assessment of plant species richness and abundance. Species richness was assessed in the second week of each month from May to August 2012. Plots were thoroughly examined and all species present were recorded. Percent occurrence of species was calculated by counting the number of plots (i.e. the 100 randomly assigned plots) containing each species. This provided an estimate of species’ abundance (i.e. percent occurrence) in our old-field site (Fig. 1).

The survey comprised four exotic and four native plant species (Table 1). In each category, i.e. exotic and native, two species were abundant and two were relatively rare (termed abundant and rare hereon) at this old-field site (Fig. 1). The species were chosen according to their natural relative abundance at the old-field site and, as such, the lack of a balanced phylogenetic design was unavoidable. Exotic grasses dominate the site and we selected two of the most dominant for our exotic abundant category (i.e. *Dactylis glomerata* and *Bromus inermis*). For the native-abundant category we selected the most abundant natives at the site (i.e. *Solidago canadensis* and *Phalaris arundinacea*). Rare (i.e. relatively rarer) species were chosen based on the presence of at least four replicates in the field. *Potentilla recta* and *Hypericum perforatum* were the selected rare exotic species and *Symphyotrichum lanceolatum* and *Rudbeckia hirta* the rare natives. Our main goal was not to control for phylogenetic relatedness or functional group but instead to survey and compare plant species according to their relative abundance in this community. Our eight selected species represent 24 % of all the species in the community. Across the entire old-field site four individuals per species were sampled for roots and to minimize potential spatial variability, sets of replicates (one individual per plant species) were sampled in close proximity to each other in a smaller area wherever possible. To account for this possible spatial variation we included the factor ‘replicate’ in our statistical model. The choice of four replicates is consistent with other studies investigating belowground fungal community interactions with plants (Johnson *et al*. 2004; Martínez-García *et al*. 2011).

**Table 1.** Plant species from our LTERS investigated in this study.

Native rare	Native abundant	Exotic rare	Exotic abundant
*R. hirta*	*S. canadensis*	*H. perforatum*	*B. inermis*
*S. lanceolatum*	*P. arundinacea*	*P. recta*	*D. glomerata*

### 454 Pyrosequencing of fungal communities

To determine the fungal community in the selected species, plants were collected including their whole-root system using a spade within 2 days in the second half of August 2013. They were placed into Ziploc bags and cooled immediately in a cold box and later at 4 °C until further processing. Plants were stored at 4 °C no longer than 24 h. Root systems were washed in the laboratory with cold tap water to remove the soil and to separate any root fragments that were not connected to the main system and which could belong to a different plant.

DNA was extracted from six technical replicates per individual plant to ensure having a representative amount of DNA. For each technical replicate twenty-one 0.5-cm-long root pieces were excised. For very thick roots, the amount of root material was adjusted accordingly to avoid overloading the DNA extraction column. Roots were surface sterilized for 30 s in 30 % hydrogen peroxide and the reaction was stopped using 70 % EtOH. They were then frozen at −80 °C and freeze-dried for 4 days (Labconco FreeZone 2.5, USA). To verify proper surface sterilization three 0.5-cm root pieces of each individual plant were excised and surface sterilized as described above and incubated at room temperature on potato dextrose agar plates. Surface sterilization of the plant root systems was successful as fungi started growing out of the roots only after several days at 20 °C. This means organisms attached to the surface were excluded but fungi within the roots were still actively growing.

DNA was extracted using the DNeasy 96 Plant Kit (Qiagen, Toronto, ON, Canada) according to the manufacturer's protocol. The extracted DNA was cleaned and purified using the Genomic DNA Clean & Concentrator™ Kit (Zymo Research, USA). After DNA purification, the six technical replicates coming from one single root system were pooled together leading to 32 samples in total (eight plant species × four replicates). The amount of DNA was measured using a BioTek™ Epoch™ Microplate Spectrophotometer (Winooski, VT, USA).

Primers fITS7 as a forward (5′-GTGARTCATCGAATCTTTG-3′) and ITS4 as a reverse (5′-TCCTCCGCTTATTGATATGC-3′) were chosen to sequence samples using 454 pyrosequencing (Ihrmark *et al*. 2012). We specifically chose the new highly specific fungal primer fITS7 because we were interested in amplifying the entire fungal community present within the plant roots. In combination with ITS4 this pair specifically amplifies the ITS 2 region and produces shorter amplicons compared with the ITS1f/ITS4 primer pair. We expected that as we sequenced an entire fungal community any bias resulting from different sequence lengths would be minimized by amplifying the shorter ITS 2 region instead of the entire internal transcribed spacer (ITS) region. According to a BLASTN search, our selected primer pair should match 68 % of the Glomeromycota (Ihrmark *et al*. 2012). Samples were sent to Genome Québec (Montréal, Canada) and purified and diluted 1 : 4 using a MultiScreen PCR filtration plate (Millipore, Canada). PCR with primers fITS7 and ITS4 was conducted in 25 µL reaction volume containing 2.5 µL purified DNA, 2.5 µL 10× buffer, 1.5 mM MgCl_2_, 0.2 µM dNTPs, 0.4 µM of each primer and 5 U µL^−1^ HotStarTaq DNA Polymerase (Qiagen) as final concentrations. PCR running conditions were as follows: initial denaturation at 96 °C for 15 min followed by 35 cycles of denaturation at 96 °C for 30 s, annealing at 52 °C for 30 s, extension at 72 °C for 1 min and a final extension step at 72 °C for 10 min. Samples were tagged using 10-bp multiplex identifiers (MID Tags) in a separate barcoding PCR reaction. The tagged, equimolar amplicons were sequenced on a Roche 454 GS FLX instrument using the FLX+ chemistry at Genome Québec. A total of 327 867 single reads were produced in a one-fourth region of a 454 plate. Sequence data were submitted to NCBI (accession no. SRP043133).

### Symbiotic relationships

To investigate symbiotic (i.e. functional) relationships between mutualistic and antagonistic belowground biota and our target species, plants were collected at our old-field site in mid-August 2012, with the exception of *R. hirta*, which could not be found on that occasion. Four individual plants were collected for each species using a spade (except for *H. perforatum* for which we could only sample three individuals). In the laboratory, root samples were carefully washed and divided into two sub-samples. One sub-sample was used to quantify the percentage of root colonization by arbuscular mycorrhizal fungi (AMF) using the gridline intersect method by McGonigle *et al*. (1990). The roots were stained using 0.03 % Chlorazol-black E and a minimum of 100 intersections per replicate were examined. The other sub-sample was used to quantify the incidence of disease and enemy attack by visually estimating damage (i.e. the number of lesions per root) caused by pathogens, parasites and herbivores. This method is identical to the root staining and quantification method used for AMF, excluding the staining step. This approach provides a consistent estimate of plant tolerance against pathogens and other enemies (Mitchell 2003; Schnitzer *et al*. 2011). For both AMF and lesion quantification, *H. perforatum* samples had to undergo an additional bleaching step using alkaline hydrogen peroxide to remove root pigmentation to allow visualization.

### Bioinformatics and statistical analysis

Bioinformatics were performed at Genome Québec using Qiime (version 1.7.0) the pipeline for data processing. In total, 327 867 reads were generated, assessed for their quality and clustered. Reads with a Phred quality score ≥30, <3 undetermined bases and <4 bases having a Phred quality score <20 passed the quality control step. All reads were cut to a final length of 220 bases. Reads were clustered at 100 % ID, followed by 99 % and finally by 97 %. Chimera clusters were removed during this step. Sequences were blasted against the ITS2 database of the UNITE ITS database (Kõljalg *et al*. 2013), trimmed to the ITS2 region. An Operational Taxonomic Units (OTU) table was generated representing fungi only and samples were rarefied to 1000 reads per sample. The Qiime pipeline (version 1.7.0) was used to calculate the rarefraction curves. After rarefaction, singletons and doubletons were removed and the resulting OTU table was used for further analysis.

To determine whether fungal communities differed between the factors ‘abundance’ (i.e. rare and abundant plant species) and ‘origin’ (i.e. native and exotic plant species), a Permutational MANOVA was conducted using function ‘adonis’ from the *vegan* package (Oksanen *et al*. 2013) implemented in the software R (R Core Team 2013). The effect of plant abundance and origin was calculated by measuring the effect of each factor after accounting for the variance explained by the other factor (variance partitioning (Schmid *et al*. 2002)). This was done after first accounting for our four replicates (i.e. spatial variability) and we also took into consideration the two different plant functional groups (i.e. monocotyledons and dicotyledons). We first ran a model including ‘plant species’ as a third factor and, as expected, plant species were a significant predictor of fungal community composition. By this factor, 42 % of the variance was explained. However, since our main goal was to survey general patterns of fungal diversity rather than species-specific effects, we considered species as ‘plant functional group’ thereby gaining degrees of freedom in our two factor model.

To calculate the variance explained by the factor ‘abundance’ (full model including interaction) we used the following formula:

(1) $$\begin{array}{rr} & \text{Dissimilarity}\;\text{matrix}∼\text{replicate}+\text{plant}\;\text{functional}\;\text{group}\\ & \;+\text{origin}+\text{abundance}+\text{origin:abundance} \end{array}$$

To calculate the variance explained by the factor ‘origin’ (full model including interaction) we used the following formula:

(2) $$\begin{array}{rr} & \text{Dissimilarity}\;\text{matrix}∼\text{replicate}+\text{plant}\;\text{functional}\;\text{group}\\ & \;+\text{abundance}+\text{origin}+\text{abundance:origin} \end{array}$$

The plant functional group was kept in the model since it explained 9.21 % of the total variance. By keeping it, the variance from this factor could be deducted from our variables of interest (‘abundance’ and ‘origin’). As dissimilarity measure, Bray–Curtis was used to account for the composition of the fungal communities as well as to exclude joint absences. α Diversity was tested for each sample using the function ‘rarefy’ from the *vegan* package (Oksanen *et al*. 2013) in R (R Core Team 2013) and an ANOVA was run to test for significant differences depending on the factors ‘abundance’ and ‘origin’. A distance matrix was created using the *vegan* package (Oksanen *et al*. 2013) in R (R Core Team 2013) to estimate β diversity using the function ‘betadisper’ and to draw principle coordinates analysis (PCoA) plots.

Another ANOVA was run to test whether AMF root colonization and lesions varied according to ‘abundance’ and ‘origin’. To meet the requirements of the statistical tests, variables were arcsine transformed. Means among treatments were compared using Tukey's honestly significant difference (HSD) test (*P* < 0.05). These statistical analyses were performed with the software StatSoft, Inc. (2013), STATISTICA (data analysis software system), version 12. www.statsoft.com.

## Results

### Fungal community patterns

**Figure 2.**
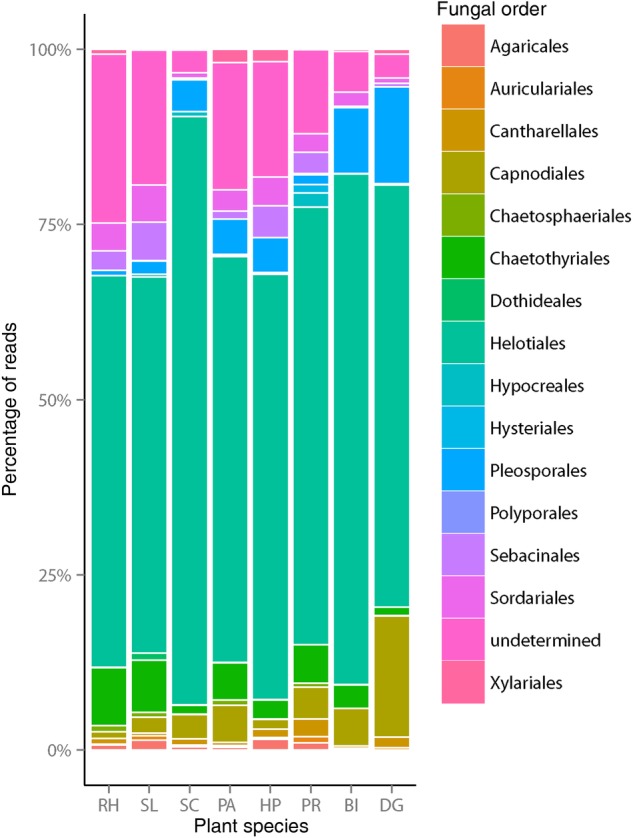
Rarified abundance of fungal sequence reads discriminated by fungal order for each plant species. ‘Undetermined’ means OTUs not determined to the level ‘Order’ but at least to the kingdom of fungi. RH, *R. hirta*; SL, *S. lanceolatum*; SC, *S. canadensis*; PA, *P. arundianacea*; HP, *H. perforatum*; PR, *P. recta*; BI, *B. inermis*; DG, *D. glomerata*.

We detected 164 fungal OTUs across all samples. The phylum Ascomycota comprised 138 OTUs (84 % of fungal OTUs) whereas Basidiomycota only comprised 23 OTUs (14 % of fungal OTUs), the remaining three OTUs (1.8 % of fungal OTUs) could only be assigned to Kingdom Fungi (Table 2). We were unable to recover AM fungi as we expected based on our primer choice. Fungal richness of rarefied and non-rarefied samples for each plant species is provided in Table 3. The two plant species *H. perforatum* and *P. arundinacea* had the highest number of fungal OTUs (Table 3) and the Helotiales was the most common fungal order across all plant species (Fig. 2 and **Supporting Information—Table S1** for detailed taxonomic results).

**Table 2.** Taxonomic fungal groups detected in the roots of all plant species in the filtered and rarefied OTU table excluding singletons and doubletons.

Taxonomic group	No. of OTUs	OTUs in %	No. of reads	Reads in %
Ascomycetes	138	84	26 754	95.5
Helotiales	57	41.3	17 964	67.1
Chaetothyriales	25	18.1	1291	4.8
Pleosporales	17	12.3	1522	5.7
Capnodiales	9	6.5	1522	5.7
Sordariales	7	5.1	809	3
Hypocreales	3	2.2	129	0.5
Xylariales	2	1.4	195	0.7
Chaetosphaeriales	2	1.4	121	0.5
Dothideales	2	1.4	50	0.2
Hysteriales	1	0.7	52	0.2
Basidiomycetes	23	14	1258	4.5
Agaricales	6	26.1	198	15.7
Auriculariales	5	21.7	102	8.1
Sebacinales	3	13	630	50.1
Cantharellales	2	8.7	285	22.7
Polyporales	1	4.3	8	0.6
Undetermined (fungi)	3	1.8	3134	11.2

**Table 3.** Fungal richness per plant species for non-rarefied and rarefied samples.

Plant species	Non-rarefied	Rarefied
*R. hirta*	45.26127	27.25000
*S. lanceolatum*	78.96880	49.00000
*S. canadensis*	75.55551	44.33333
*P. arundinacea*	107.10158	64.75000
*H. perforatum*	108.59286	55.25000
*P. recta*	63.01892	40.33333
*B. inermis*	69.51156	43.50000
*D. glomerata*	64.02056	39.25000

**Figure 3.**
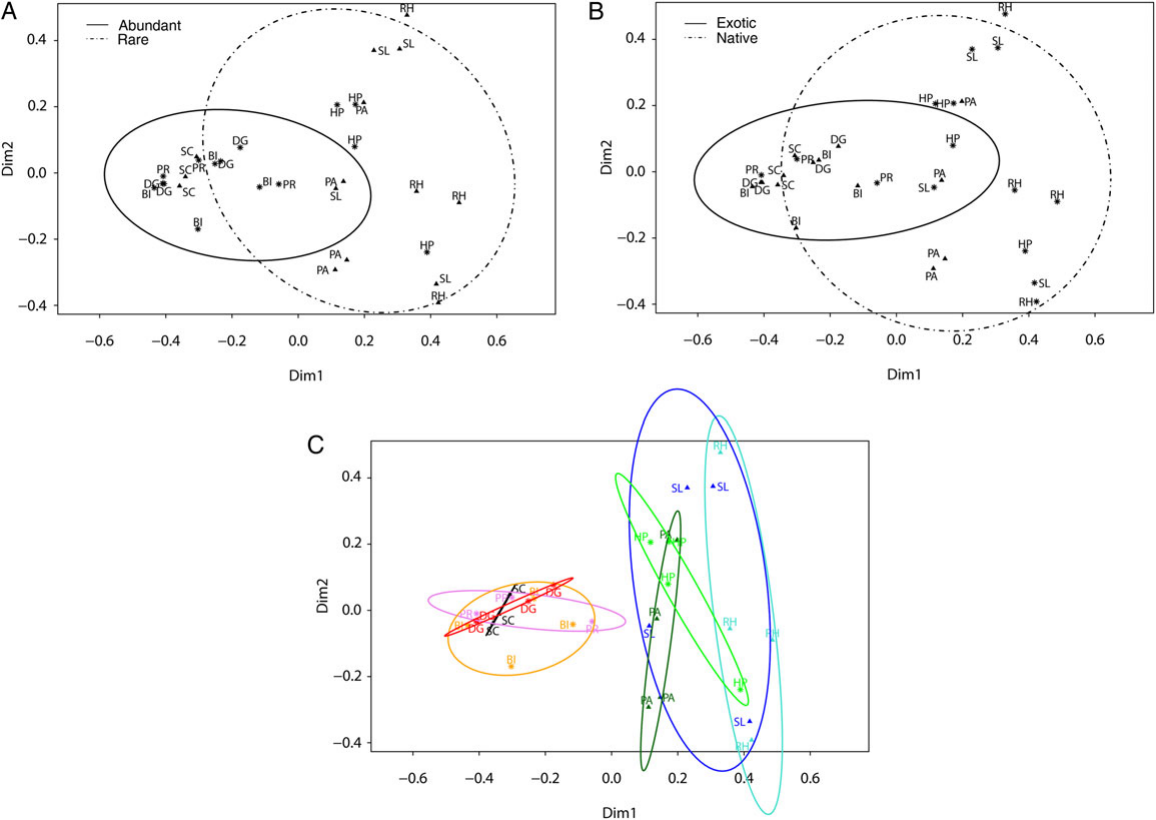
Principal coordinate analysis plots discriminating between belowground root-associated fungal communities according to (A) abundance, (B) origin and (C) plant species. In (A and C) exotic plant species are labeled with a star and native species with triangles and in (B) abundant plant species are labeled with a triangle and rare species with a star. Confidence interval = 80 % for (A and B) and 75 % for (C). RH, *R. hirta*; SL, *S. lanceolatum*; SC, *S. canadensis*; PA, *P. arundianacea*; HP, *H. perforatum*; PR, *P. recta*; BI, *B. inermis*; DG, *D. glomerata*.

Permutational MANOVA showed significant fungal community dissimilarities for ‘abundance’ (*F*_1,29_ = 5.194, *R*^2^ = 0.140, *P* = 0.001) and ‘origin’ (*F*_1,29_ = 4.590, *R*^2^ = 0.120, *P* = 0.001) after accounting for both the spatial variation (factor ‘replicate’) in our field site (*F*_3,29_ = 1.322 with *R*^2^ = 0.103, *P* = 0.161 (1) and, *R*^2^ = 0.103, *P* = 0.140 (2)) and our two plant functional groups (monocotyledons and dicotyledons) which explained 9.21 % of the total variance (*F*_1,29_ = 3.756 with *R*^2^ = 0.092, *P* = 0.005 (1) and *R*^2^ = 0.092, *P* = 0.002 (2)). However, the interaction of ‘abundance’ and ‘origin’ was not significant (*F*_1,29_ = 1.235, *R*^2^ = 0.034, *P* = 0.255) in either of the two models and therefore, we continued with the analysis using the reduced models (i.e. by excluding the interaction but keeping the factor ‘replicate’) (Table 4). The PCoA plots were in concert with the results from the permutational MANOVA pointing towards the structuring of fungal communities as a function of ‘abundance’ and ‘origin’ (Fig. 3). Figure 3C shows that our ‘plant functional groups’ did not share the same fungal communities but those were rather dependent on origin except for *H. perforatum* and *S. canadensis*.

**Table 4.** Factors and their interaction referring to community dissimilarity and resulting from the Permutational MANOVA for the response variables ‘abundance’ and ‘origin’; *Significant at *α* = 0.05.

	*F*-value	*R*^2^	*P*-value
Calculating factor ‘abundance’			
Abundance	5.194	0.14	0.001*
Replicate	1.322	0.103	0.161
Plant functional group	3.756	0.092	0.005*
Abundance × origin	1.235	0.034	0.255
Calculating factor ‘origin’			
Origin	4.59	0.12	0.001*
Replicate	1.322	0.103	0.14
Plant functional group	3.756	0.092	0.002*
Abundance × origin	1.235	0.034	0.255

**Figure 4.**
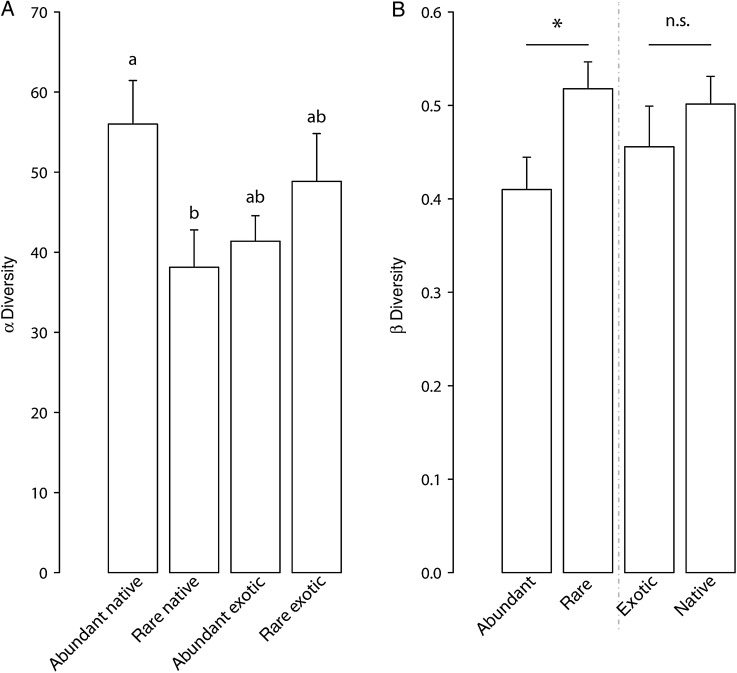
α (A) and β (B) diversity of fungal communities for the factors ‘abundance’ and ‘origin’ in plant roots from a natural old-field community. α Diversity showed a significant interaction between ‘abundance’ and ‘origin’ (*P* = 0.014). Standard errors are indicated by error bars. Means in a bar sharing the same letter are not significantly different according to Tukey's HSD at a significance level of *P*≤ 0.05.

α Diversity, i.e. the number of OTUs per sample, was not affected by the factors ‘abundance’ (*F*_1,26_ = 1.108, *P* = 0.302) or ‘origin’ (*F*_1,26_ = 0.163, *P* = 0.690) but their interaction was significant (*F*_1,26_ = 6.908, *P* = 0.014) (Fig. 4A). Fungal β diversity varied significantly depending on plant species’ relative ‘abundance’ (*F*_1,28_ = 5.77, *P* = 0.017) but not on their ‘origin’ (Fig. 4B).

Overall, abundant plant species tended to associate with a more similar fungal community compared with rare plants (Fig. 3A). This was also supported by β diversity (Fig. 4B). In addition, PCoA indicated that compared with native plants, fungal community composition among exotics was more similar (Fig. 3B).

### Symbiotic relationship patterns

**Figure 5.**
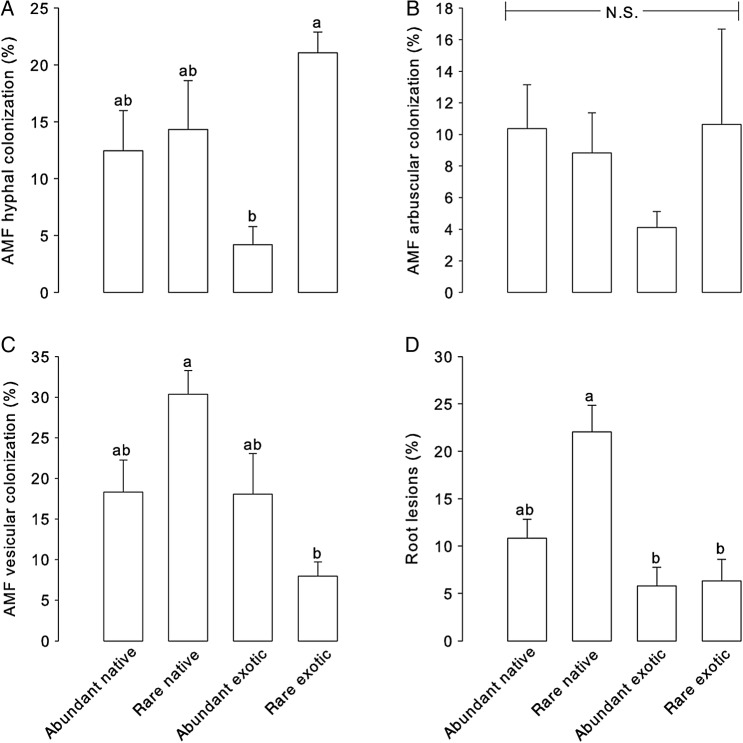
Arbuscular mycorrhizal fungal colonization by hyphae (A), arbuscules (B) and vesicles (C) and incidence of lesions (D) in the roots of native and exotic plants of contrasting relative abundance in a ruderal natural plant community. Means in a bar sharing the same letter are not significantly different according to Tukey's HSD at a significance level of *P*≤ 0.05.

Species ‘abundance’ as well as the interaction between ‘abundance’ and ‘origin’ significantly determined AMF colonization by hyphae in our selected plant groups (*F*_1,23_ = 11.548, *P* = 0.002 and *F*_1,23_ = 6.972, *P* = 0.015, respectively). Rare exotic plants had the largest amount of AMF colonization and abundant exotics the least. In contrast, hyphal colonization did not vary significantly with plant relative abundance for native plants (Fig. 5A). Arbuscular colonization showed the same trends but the results were not statistically significant due to a high variance for the rare exotic species, which were underrepresented (Fig. 5B). We found a significant effect of ‘origin’ as well as ‘origin’ × ‘abundance’ for vesicular colonization (*F*_1,23_ = 7.663, *P* = 0.011 and *F*_1,23_ = 6.937, *P* = 0.015, respectively). However, this was in contrast with hyphal colonization. The rare native species had the greatest number of vesicles and rare exotics the least (Fig. 5C).

‘Origin’ was a significant factor determining the susceptibility of plant species to enemy attack belowground (*F*_1,22_ = 17.811, *P*≤ 0.001), and despite the lower replication of rare plants due to the absence of *R. hirta*, we detected a marginally significant effect of ‘abundance’ (*F*_1,22_ = 4.148, *P* = 0.054). In addition, there was a marginally significant effect for the interaction (*F*_1,22_ = 3.273, *P* = 0.084)*.* Rare natives were the most susceptible group to enemy attack whereas for exotics, their lesion number was the same regardless of their relative abundance in the community (Fig. 5D).

## Discussion

The main objective of this study was to investigate patterns connected with plant and belowground fungal community assembly. More specifically, we determined whether plant species of contrasting relative abundance and geographic origin in a natural old-field community typical of eastern North America associated differently with the belowground fungal community. Our main goal was to primarily survey plants based on their relative abundance. As such, the lack of a balanced phylogenetic design was an unavoidable feature of the study system. Our choice of plant species provides insight into how natural plant diversity may contribute to influence the belowground fungal community of a typical old-field grassland in eastern North America. Nevertheless, in addition to plant abundance (abundant and rare) and plant origin (native and exotic) we accounted for the proportion of the variance explained by plant functional groups (monocotyledons and dicotyledons). Our results were consistent with our prediction that fungal communities associated differently with plant species depending on the plants’ relative ‘abundance’ and ‘origin’.

Plant species ‘abundance’ was one of the two factors influencing fungal communities in plant roots suggesting that soil communities may also play a role in determining the relative abundance of our plant species in the community. This is consistent with reports emphasizing the importance of soil microbial communities in structuring plant communities (Klironomos 2002; Bever *et al*. 2010; Pendergast *et al*. 2013).

Partly consistent with our prediction, we found β diversity to be significantly higher for rare than for abundant plant species, indicating that fungal communities among abundant plant species were more similar compared with those present in rare plant species (Fig. 4B). This result was additionally supported by the PCoA plot (Fig. 3A). However, the interaction between ‘abundance’ and ‘origin’ was not significant contrary to our prediction that patterns of fungal community diversity were connected to both plant abundance and origin. β Diversity was not significantly different between exotic and native species. β Diversity of rare plants may be influenced by the fungal community of neighboring plant species which are likely to be composed of different plant species. Conversely, abundant plants are more often surrounded by conspecifics and therefore, share a more similar fungal community than rare plant species. Alternatively, our four rare species (Table 1) may be more selective in their fungal associations, explaining the higher overall β diversity but lower α diversity. Another scenario could be that rare species occupy a greater number of microsites to avoid competition as they are presumably rather weak competitors compared with abundant plant species. Due to these different environments rare plants cannot be very selective in choosing their fungal communities. This in turn might influence their β diversity which was rather high compared with abundant species. Since rarity in communities may be driven by negative feedback (Klironomos 2002) based on our results it is tempting to speculate that rare native species may associate with putatively more antagonistic fungi (Bever *et al*. 2012; Van der Putten *et al*. 2013). This was further supported by our root lesion data, showing that root enemies affected mostly rare native plants (Fig. 5B). Additionally, a negative feedback has been shown to be prevalent in grasslands maintaining a high plant diversity (Kulmatiski *et al*. 2008).

Fungal richness (α diversity) in abundant native plants was higher than in rare natives but for exotics we did not observe a significant difference (Fig. 4A). Perhaps such higher fungal diversity in the roots of native plants could be important to explain their abundance. Ecosystems and communities may be considered to be more stable if biodiversity is higher since productive species can compensate for other species that may not perform as well (Naeem and Li 1997). This may also be linked to the success of individual plant species. For instance, higher genotypic diversity of root-associated dark septate endophytic fungi has been shown to increase their host plants’ growth rate (Reininger *et al*. 2012). Perhaps the microbial communities associated with abundant native plant species may be even more conducive to positive feedback (Van der Putten *et al*. 2013).

As predicted, fungal communities associating with exotic species were different from fungal communities associating with native species. However, we found no differences in α- and β diversity between abundant exotic and rare exotic species, and exotic and native species, respectively. This suggests that the relative abundance of exotics in the community may be independent from their fungal communities. Since exotic species may have escaped from their co-evolved soil enemies (Keane and Crawley 2002; Agrawal *et al*. 2005), they might be uncoupled from the requirement to associate with a specific belowground microbial community. Despite the importance of fungi as drivers of plant community structure (Van der Heijden *et al*. 1998; Reynolds *et al*. 2003) only very few studies have compared the fungal communities associating with exotics versus natives in a natural community (Bongard *et al*. 2013). Consistent with our results, Nelson and Karp (2013) found that distinct communities of pathogenic fungi associate with native and exotic haplotypes of *Phragmites australis*, suggesting that exotic plants may associate with a different fungal community than native species.

Our molecular methodological approach detected fungi belonging to the Ascomycota, which is the largest clade of fungi (Kirk *et al*. 2001; Wang *et al*. 2006), and Basidiomycota. More specifically, the most represented order across all plant species regardless of their ‘abundance’ and ‘origin’ were the Helotiales belonging to the Ascomycota (Fig. 2). This very diverse and large fungal order includes ∼2300 species which take several ecological functional roles ranging from ericoid mycorrhiza, endophytes, saprobes and pathogens (Webster and Weber 2007). Therefore, it is not surprising that we found so many OTUs belonging to this large and diverse fungal order. Since OTUs were not determined to species level, we were unable to make any statement about their particular function in our old-field site.

The ITS region has been shown a high degree of success for the broad detection of fungi (Buée *et al*. 2009) and was recently proposed to serve as the primary fungal barcode marker (Schoch *et al*. 2012). Even though the primer pair fITS7/ITS4 has been tested positively to match Glomeromycota (Ihrmark *et al*. 2012), we failed to do so even though AMF were certainly present in the roots of our plant species (Fig. 5A). This is in contrast with a BLASTN search at NCBI with fITS7, which matched 68 % of the Glomeromycota 5.8S sequences (Ihrmark *et al*. 2012). Even though we selected this primer pair taking into consideration the shorter sequence length, it is known that the choice of ITS could bias against certain groups such as Early Diverging Lineages, Ascomycota and Glomeromycota (see Schoch *et al*. 2012). For the latter group, Stockinger *et al*. (2010) proposed a larger barcode, combining the ITS and LSU regions. Furthermore, due to the fact that in our study the primers were degenerate, there is an increasing chance of getting unspecific results by running too many PCR cycles (Ihrmark *et al*. 2012). This primer bias can be expected as a result of a high number of PCR cycles (35) and the low GC content of the Glomeromycota (Stockinger *et al*. 2010; Gianinazzi-Pearson *et al*. 2012; Kauserud *et al*. 2012; Pinto and Raskin 2012). Nevertheless, all our samples were treated equally and even though we were unable to capture the full breadth of the fungal Kingdom, our 454 pyrosequencing approach was able to consistently detect a broad diversity of taxa among our plant groups. This is in accordance with Ihrmark *et al*. (2012) as our primers are supposed to better preserve the quantitative composition of the template between-sample differences in fungal community composition. We also need to consider that fungal community composition shifts seasonally (Thoms and Gleixner 2013; Voříšková *et al*. 2014). The sampling was undertaken mid-summer to ensure that fungal communities had sufficient time to re-establish after the winter. However, our study represents a snapshot of the fungal community at a certain time. As such it is entirely possible that the outcome of the study might be different in another season and it would be interesting to contrast our results with those collected in late fall or early spring. Furthermore, our study should be seen as a first coarse survey of fungal communities across a selected number of plants of contrasting abundance and origin in a community. Future work should include more species in this and other communities and take into consideration phylogenetic relationships.

We were able to evaluate patterns of root interactions with mutualistic and antagonistic soil biota by estimating AM fungal colonization and lesions in plant roots, respectively (Fig. 5). Arbuscular mycorrhizal fungi are considered widespread among plants and represent perhaps the most important mutualism globally (Van der Heijden *et al*. 1998; Wang and Qiu 2006; Parniske 2008; Öpik *et al*. 2010; Kiers *et al*. 2011). We found that AMF barely colonized the most abundant exotic plants (i.e. <5 %), which could be considered as invasive plants in our old-field site (Fig. 1). These plants also happened to be grasses and some subfamilies in the Poaceae (e.g. subfamily Pooideae to which *B. inermis* belongs) are known to have low mycorrhizal responsiveness and colonization (Reinhart *et al*. 2012). The opposite was true for rare exotic species (Fig. 5A). Taken together, our results are consistent with the framework proposed by Pringle *et al.* (2009) suggesting, despite the very limited data available, that at least for North America, exotic invasive plants (i.e. in this particular case, mostly grasses in old-field systems) may not associate or depend on AMF. Taking this into consideration, our results are consistent with studies showing that abundant cool-season grasses have low mycorrhizal dependency (i.e. their biomass responses are not correlated with AMF colonization (Reinhart *et al*. 2012; Treseder 2013) whereas exotic forbs can establish strong associations with AMF (Wilson and Hartnett 1998; Lekberg *et al*. 2013).

Our results showed that rare native plants harbored more vesicles, which are energy reserve structures and whose production may be triggered in response to stress (Smith and Read 2008) (Fig. 5C). They were also significantly more affected by root enemies than exotic plants present in the community (Fig. 5D). This confirms and further provides a mechanism (i.e. increased specialist plant biotic interactions) for the result of a previous greenhouse feedback study with soil biota from an old-field plant community in which negative feedback was stronger on rare native plants (Klironomos 2002). It is possible that some of the fungal groups detected using our sequencing approach may be responsible for some of the observed root lesions present in higher number on native rare plants. However, considering that fungal function is highly context dependent (e.g. host dependent), future work should use a more targeted approach that can provide higher taxonomic resolution and capture functional processes, to determine the extent to which changes in fungal diversity associating with roots are either beneficial or detrimental to particular plants species or guilds.

To our knowledge, this is the first study investigating linkages between belowground fungal community structure and abundant and rare plant species in a natural grassland community, including plant species originating from different geographic origins. Future research connected to this field should use molecular methodologies capable to increase the level of taxonomic resolution. In addition, a major goal should be the establishment of links between taxonomic and functional diversity driving plant communities.

## Conclusions

In conclusion, our results are the first linking natural plant abundance and geographic origin with fungal community structure. Overall, plant relative abundance but not origin determined fungal β diversity. Fungal α diversity varied only for native species of contrasting abundance; not for exotics. Taken together, this suggests the structuring of fungal communities even in the remnant native plants of an invaded community. Conversely, exotic plants appear to act as a disturbing (homogenizing) agent of the fungal community possibly changing feedback interactions across the entire plant community (e.g. mutualist disruption). These patterns may be linked to invasion meltdown processes and need further investigation.

## Accession Number

Sequence data was submitted to NCBI (accession no. SRP043133).

## Sources of Funding

This study was funded by a Research Chair from the Ontario Ministry of Natural Resources and by a Natural Sciences and Engineering Research Council of Canada (NSERC) Discovery Grant awarded to P.M.A.

## Contributions by the Authors

P.M.A. originally formulated the idea, P.M.A., V.R. and L.S. developed original idea, V.R. and L.S. developed methodology, V.R., L.S. and P.M.A. conducted fieldwork, V.R. generated sequencing data and molecular analyses, V.R., L.B.M.-G. and P.M.A. performed statistical analyses, and V.R., L.B.M.-G., P.M.A. and L.S. wrote the manuscript.

## Conflict of Interest Statement

None declared.

## Supporting Information

The following additional information is available in the online version of this article –

**Table S1.** Detailed taxonomic information of OTUs obtained in this study using 454 pyrosequencing.

Additional Information
