# Supplementary material for: Composition of fungal soil communities varies with plant abundance and geographic origin
Source: AoB Plants. 2015 Sep 14;7:plv110. doi: 10.1093/aobpla/plv110 (PMC4614812; doi:10.1093/aobpla/plv110)
Supplement: Additional Information [file supp_7_plv110_index.html]

Composition of fungal soil communities varies with plant abundance and geographic origin — Composition of fungal soil communities varies with plant abundance and geographic origin — Additional Information 

# Composition of fungal soil communities varies with plant abundance and geographic origin

## Additional Information

Additional Information

- Supplementary Table - xlsx file
